# (*E*)-1-(3-Methoxy­phen­yl)ethanone 4-nitro­phenyl­hydrazone

**DOI:** 10.1107/S1600536808018618

**Published:** 2008-06-25

**Authors:** Zheng Fan, Shang Shan, Shan-Heng Wang, Wen-Long Wang

**Affiliations:** a College of Biological and Environmental Engineering, Zhejiang University of Technology, People’s Republic of China; b College of Chemical Engineering and Materials Science, Zhejiang University of Technology, People’s Republic of China

## Abstract

Crystals of the title compound, C_15_H_15_N_3_O_3_, were obtained from a condensation reaction of 4-nitro­phenyl­hydrazine and 3-methoxy­acetophenone. In the crystal structure, the methoxy­phenyl ring is twisted slightly with respect to the nitro­phenyl­hydrazine plane, making a dihedral angle of 14.81 (8)°. The nitro and meth­oxy groups are each coplanar with the attached benzene rings. The nitro­phenyl and methoxy­phenyl groups are located on opposite sides of the C=N double bond, indicating an *E* configuration of the mol­ecule. Adjacent mol­ecules are linked together *via* N—H⋯O hydrogen bonding, forming chains along the [101] direction.

## Related literature

For general background, see: Okabe *et al.* (1993 ▶); Shan *et al.* (2003*a* ▶). For related structures, see: Shan *et al.* (2003*b* ▶, 2004 ▶, 2008 ▶).
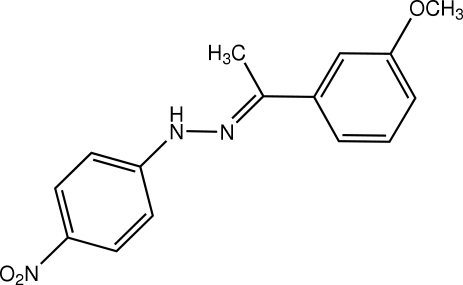


## Experimental

### 

#### Crystal data

C_15_H_15_N_3_O_3_
*M* *_r_* = 285.30
Monoclinic, 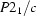

*a* = 4.2977 (17) Å
*b* = 24.709 (9) Å
*c* = 13.132 (5) Å
β = 96.332 (11)°
*V* = 1386.0 (9) Å^3^
*Z* = 4
Mo *K*α radiation
μ = 0.10 mm^−1^
*T* = 295 (2) K
0.32 × 0.26 × 0.22 mm

#### Data collection

Rigaku R-AXIS RAPID IP diffractometer
Absorption correction: none
16470 measured reflections
3014 independent reflections
1643 reflections with *I* > 2σ(*I*)
*R*_int_ = 0.045

#### Refinement

*R*[*F* ^2^ > 2σ(*F* ^2^)] = 0.049
*wR*(*F* ^2^) = 0.141
*S* = 1.03
3014 reflections
192 parameters
H-atom parameters constrained
Δρ_max_ = 0.15 e Å^−3^
Δρ_min_ = −0.17 e Å^−3^

### 

Data collection: *PROCESS-AUTO* (Rigaku, 1998 ▶); cell refinement: *PROCESS-AUTO*; data reduction: *CrystalStructure* (Rigaku/MSC, 2002 ▶); program(s) used to solve structure: *SIR92* (Altomare *et al.*, 1993 ▶); program(s) used to refine structure: *SHELXL97* (Sheldrick, 2008 ▶); molecular graphics: *ORTEP-3 for Windows* (Farrugia, 1997 ▶); software used to prepare material for publication: *WinGX* (Farrugia, 1999 ▶).

## Supplementary Material

Crystal structure: contains datablocks I, global. DOI: 10.1107/S1600536808018618/om2241sup1.cif

Structure factors: contains datablocks I. DOI: 10.1107/S1600536808018618/om2241Isup2.hkl

Additional supplementary materials:  crystallographic information; 3D view; checkCIF report

## Figures and Tables

**Table 1:** Hydrogen-bond geometry (Å, °)

*D*—H⋯*A*	*D*—H	H⋯*A*	*D*⋯*A*	*D*—H⋯*A*
N2—H2⋯O3^i^	0.86	2.45	3.279 (2)	161

Symmetry code: (i) 
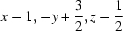
.

**Fig. 1.**
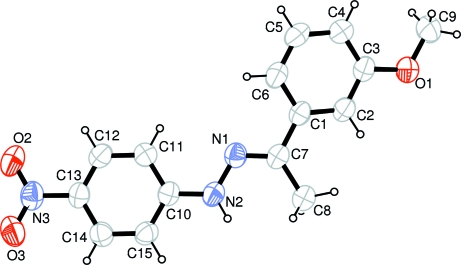
The molecular structure of the title compound with 30% probability displacement ellipsoids for non-H atoms.

**Fig. 2.**
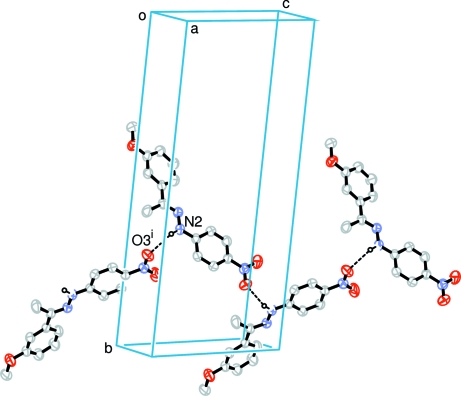
A diagram showing the N—H···O hydrogen bond chain (dashed lines) [symmetry code: (i) -1 + x, 3/2 - y, -1/2 + z].

## supplementary crystallographic information

### Comment

Since some phenylhydrazone derivatives have shown to be potential DNA damaging and mutagenic agents (Okabe *et al.*, 1993), a series of new phenylhydrazone derivatives have been prepared in our laboratory (Shan *et al.*, 2003*a*). As part of the ongoing investigation, the title compound has recently been prepared and its crystal structure is reported here.

The molecular structure of the title compound is shown in Fig. 1. The N1—C7 bond distance of 1.295 (2) Å indicates a typical C=N double bond. The molecule assumes an E configuration, with the nitrophenyl ring and methoxyphenyl rings located on the opposite sites of the C=N bond. The dihedral angle of 1.4 (3)° between nitro group and C10-benzene ring and the C1—C2—C3—C4 torsion angle of 0.9 (3)° suggest that nitro and methoxyl groups are co-planar with the individual benzene rings. The methoxyphenyl ring is slightly twisted with respect to the nitrophenylhydrazine mean plane by a small dihedral angle of 14.81 (8)°, indicating the molecule is approximately co-planar except for methyl H atoms.

In the crystal structure adjacent molecules are linked *via* N—H···O hydrogen bonding to form chains along the [1 0 1] direction (Table 1 and Fig. 2). Although π-π stacking was found between 4-nitrophenyl rings in several related structures previously reported, benzil 4-nitrophenylhydrazone (Shan *et al.*, 2003*b*), 2-chloro-3,4-dimethoxybenzaldehyde 4-nitrophenylhydrazone (Shan *et al.*, 2004) and acetylpyrazine 4-nitrophenylhydrazone (Shan *et al.*, 2008), no π-π stacking is observed in the crystal structure.

### Experimental

4-Nitrophenylhydrazine (0.31 g, 2 mmol) was dissolved in ethanol (10 ml), then H_2_SO_4_ solution (98%, 0.5 ml) was added slowly to the ethanol solution with stirring. The solution was heated at about 333 K for several minutes until the solution cleared. An ethanol solution (5 ml) of 3-methoxyacetophenone (0.30 g, 2 mmol) was dropped slowly into the above solution with continuous stirring, and the mixture solution was kept at about 333 K for 0.5 h. When the solution had cooled to room temperature, red microcrystals appeared. They were separated and washed with cold water three times to get the product 0.45 g. Single crystals of the title compound were obtained by recrystallization from an absolute ethanol solution.

### Refinement

Methyl H atoms were placed in calculated positions with C—H = 0.96 Å and the torsion angle was refined to fit the electron density, *U*_iso_(H) = 1.5*U*_eq_(C). Other H atoms were placed in calculated positions with C—H = 0.93 and N—H = 0.86 Å, and refined in riding mode with *U*_iso_(H) = 1.2*U*_eq_(C,*N*).

### Crystal data

**Table:**

C_15_H_15_N_3_O_3_	*F*_000_ = 600
*M**_r_* = 285.30	*D*_x_ = 1.367 Mg m^−^^3^
Monoclinic, *P*2_1_/*c*	Mo *K*α radiation λ = 0.71073 Å
Hall symbol: -P 2ybc	Cell parameters from 4665 reflections
*a* = 4.2977 (17) Å	θ = 2.0–25.0º
*b* = 24.709 (9) Å	µ = 0.10 mm^−^^1^
*c* = 13.132 (5) Å	*T* = 295 (2) K
β = 96.332 (11)º	Prism, red
*V* = 1386.0 (9) Å^3^	0.32 × 0.26 × 0.22 mm
*Z* = 4	

### Data collection

**Table:**

Rigaku R-AXIS RAPID IP diffractometer	3014 independent reflections
Radiation source: fine-focus sealed tube	1643 reflections with *I* > 2σ(*I*)
Monochromator: graphite	*R*_int_ = 0.045
Detector resolution: 10.00 pixels mm^-1^	θ_max_ = 27.0º
*T* = 295(2) K	θ_min_ = 1.7º
ω scans	*h* = −5→5
Absorption correction: none	*k* = −30→31
16470 measured reflections	*l* = −16→15

### Refinement

**Table:**

Refinement on *F*^2^	Secondary atom site location: difference Fourier map
Least-squares matrix: full	Hydrogen site location: inferred from neighbouring sites
*R*[*F*^2^ > 2σ(*F*^2^)] = 0.049	H-atom parameters constrained
*wR*(*F*^2^) = 0.141	*w* = 1/[σ^2^(*F*_o_^2^) + (0.0685*P*)^2^ + 0.02*P*] where *P* = (*F*_o_^2^ + 2*F*_c_^2^)/3
*S* = 1.03	(Δ/σ)_max_ < 0.001
3014 reflections	Δρ_max_ = 0.15 e Å^−^^3^
192 parameters	Δρ_min_ = −0.16 e Å^−^^3^
Primary atom site location: structure-invariant direct methods	Extinction correction: none

### Special details

**Table:**

Geometry. All e.s.d.'s (except the e.s.d. in the dihedral angle between two l.s. planes) are estimated using the full covariance matrix. The cell e.s.d.'s are taken into account individually in the estimation of e.s.d.'s in distances, angles and torsion angles; correlations between e.s.d.'s in cell parameters are only used when they are defined by crystal symmetry. An approximate (isotropic) treatment of cell e.s.d.'s is used for estimating e.s.d.'s involving l.s. planes.
Refinement. Refinement of *F*^2^ against ALL reflections. The weighted *R*-factor *wR* and goodness of fit *S* are based on *F*^2^, conventional *R*-factors *R* are based on *F*, with *F* set to zero for negative *F*^2^. The threshold expression of *F*^2^ > σ(*F*^2^) is used only for calculating *R*-factors(gt) *etc*. and is not relevant to the choice of reflections for refinement. *R*-factors based on *F*^2^ are statistically about twice as large as those based on *F*, and *R*- factors based on ALL data will be even larger.

### Fractional atomic coordinates and isotropic or equivalent isotropic displacement parameters (Å^2^)

**Table:**

	*x*	*y*	*z*	*U*_iso_*/*U*_eq_	
N1	0.2858 (3)	0.59466 (6)	0.29114 (11)	0.0540 (4)	
N2	0.4535 (3)	0.63991 (6)	0.27160 (11)	0.0573 (4)	
H2	0.4405	0.6533	0.2109	0.069*	
N3	1.2264 (4)	0.73954 (7)	0.58666 (13)	0.0656 (5)	
O1	−0.4958 (3)	0.41329 (6)	0.10899 (10)	0.0775 (5)	
O2	1.2533 (4)	0.71902 (6)	0.67268 (11)	0.0944 (6)	
O3	1.3617 (4)	0.78225 (6)	0.56823 (11)	0.0865 (5)	
C1	−0.0634 (4)	0.52365 (7)	0.24122 (12)	0.0507 (5)	
C2	−0.2090 (4)	0.48991 (7)	0.16601 (13)	0.0563 (5)	
H2A	−0.2011	0.4985	0.0974	0.068*	
C3	−0.3663 (4)	0.44357 (8)	0.19125 (13)	0.0574 (5)	
C4	−0.3852 (5)	0.43034 (8)	0.29148 (15)	0.0680 (6)	
H4	−0.4900	0.3993	0.3087	0.082*	
C5	−0.2442 (5)	0.46436 (9)	0.36672 (14)	0.0775 (7)	
H5	−0.2576	0.4560	0.4351	0.093*	
C6	−0.0856 (5)	0.51000 (8)	0.34328 (14)	0.0669 (6)	
H6	0.0075	0.5319	0.3955	0.080*	
C7	0.1106 (4)	0.57271 (7)	0.21575 (13)	0.0528 (5)	
C8	0.0787 (5)	0.59459 (9)	0.10844 (15)	0.0818 (7)	
H8A	0.0396	0.6328	0.1099	0.123*	
H8B	−0.0926	0.5769	0.0686	0.123*	
H8C	0.2686	0.5880	0.0782	0.123*	
C9	−0.6460 (5)	0.36382 (8)	0.13053 (16)	0.0790 (7)	
H9A	−0.5045	0.3419	0.1746	0.119*	
H9B	−0.7058	0.3448	0.0677	0.119*	
H9C	−0.8290	0.3715	0.1638	0.119*	
C10	0.6430 (4)	0.66358 (7)	0.35048 (13)	0.0488 (4)	
C11	0.6824 (4)	0.64122 (7)	0.44883 (14)	0.0574 (5)	
H11	0.5794	0.6094	0.4625	0.069*	
C12	0.8742 (4)	0.66645 (8)	0.52547 (14)	0.0582 (5)	
H12	0.8993	0.6518	0.5911	0.070*	
C13	1.0288 (4)	0.71338 (7)	0.50517 (13)	0.0520 (5)	
C14	0.9974 (4)	0.73569 (7)	0.40765 (14)	0.0566 (5)	
H14	1.1054	0.7670	0.3942	0.068*	
C15	0.8043 (4)	0.71083 (7)	0.33116 (14)	0.0564 (5)	
H15	0.7809	0.7257	0.2657	0.068*	

### Atomic displacement parameters (Å^2^)

**Table:**

	*U*^11^	*U*^22^	*U*^33^	*U*^12^	*U*^13^	*U*^23^
N1	0.0518 (9)	0.0552 (9)	0.0533 (9)	0.0029 (7)	−0.0015 (7)	−0.0024 (7)
N2	0.0604 (10)	0.0626 (10)	0.0466 (9)	−0.0004 (8)	−0.0039 (7)	0.0005 (7)
N3	0.0698 (11)	0.0610 (11)	0.0636 (11)	0.0079 (9)	−0.0042 (8)	−0.0123 (9)
O1	0.1001 (11)	0.0751 (9)	0.0548 (8)	−0.0272 (8)	−0.0025 (7)	−0.0045 (7)
O2	0.1219 (14)	0.1013 (12)	0.0540 (9)	−0.0124 (10)	−0.0175 (9)	−0.0037 (8)
O3	0.1030 (12)	0.0664 (10)	0.0860 (11)	−0.0165 (9)	−0.0083 (9)	−0.0133 (8)
C1	0.0511 (11)	0.0553 (11)	0.0446 (10)	0.0082 (9)	0.0000 (8)	−0.0006 (8)
C2	0.0628 (12)	0.0616 (12)	0.0435 (10)	0.0016 (9)	0.0011 (9)	−0.0009 (8)
C3	0.0604 (12)	0.0628 (12)	0.0472 (11)	0.0002 (10)	−0.0021 (9)	−0.0039 (9)
C4	0.0795 (15)	0.0695 (13)	0.0539 (12)	−0.0123 (11)	0.0031 (10)	0.0044 (10)
C5	0.1069 (18)	0.0822 (16)	0.0425 (11)	−0.0145 (13)	0.0039 (11)	0.0070 (10)
C6	0.0812 (15)	0.0711 (13)	0.0454 (11)	−0.0056 (11)	−0.0058 (10)	−0.0020 (9)
C7	0.0540 (11)	0.0578 (11)	0.0456 (11)	0.0077 (9)	0.0014 (9)	−0.0020 (8)
C8	0.0992 (17)	0.0880 (16)	0.0542 (12)	−0.0295 (13)	−0.0098 (11)	0.0072 (10)
C9	0.0948 (16)	0.0627 (13)	0.0760 (15)	−0.0158 (12)	−0.0063 (12)	−0.0003 (11)
C10	0.0459 (10)	0.0516 (11)	0.0481 (10)	0.0075 (8)	0.0014 (8)	−0.0032 (8)
C11	0.0610 (12)	0.0545 (11)	0.0557 (12)	−0.0037 (9)	0.0027 (9)	0.0008 (9)
C12	0.0644 (12)	0.0630 (12)	0.0465 (11)	0.0042 (10)	0.0019 (9)	0.0022 (9)
C13	0.0518 (11)	0.0514 (11)	0.0511 (11)	0.0084 (9)	−0.0020 (8)	−0.0077 (8)
C14	0.0564 (12)	0.0496 (11)	0.0625 (12)	0.0029 (9)	0.0001 (9)	−0.0003 (9)
C15	0.0601 (12)	0.0566 (11)	0.0509 (11)	0.0044 (9)	−0.0013 (9)	0.0055 (9)

### Geometric parameters (Å, °)

**Table:**

N1—C7	1.295 (2)	C6—H6	0.9300
N1—N2	1.3699 (19)	C7—C8	1.501 (3)
N2—C10	1.376 (2)	C8—H8A	0.9600
N2—H2	0.8600	C8—H8B	0.9600
N3—O2	1.232 (2)	C8—H8C	0.9600
N3—O3	1.242 (2)	C9—H9A	0.9600
N3—C13	1.443 (2)	C9—H9B	0.9600
O1—C3	1.380 (2)	C9—H9C	0.9600
O1—C9	1.425 (2)	C10—C15	1.395 (2)
C1—C2	1.388 (2)	C10—C11	1.398 (3)
C1—C6	1.395 (2)	C11—C12	1.378 (2)
C1—C7	1.482 (2)	C11—H11	0.9300
C2—C3	1.388 (2)	C12—C13	1.377 (3)
C2—H2A	0.9300	C12—H12	0.9300
C3—C4	1.367 (3)	C13—C14	1.387 (2)
C4—C5	1.385 (3)	C14—C15	1.375 (2)
C4—H4	0.9300	C14—H14	0.9300
C5—C6	1.370 (3)	C15—H15	0.9300
C5—H5	0.9300		
			
C7—N1—N2	118.18 (15)	C7—C8—H8B	109.5
N1—N2—C10	119.09 (14)	H8A—C8—H8B	109.5
N1—N2—H2	120.5	C7—C8—H8C	109.5
C10—N2—H2	120.5	H8A—C8—H8C	109.5
O2—N3—O3	121.99 (17)	H8B—C8—H8C	109.5
O2—N3—C13	118.92 (18)	O1—C9—H9A	109.5
O3—N3—C13	119.08 (17)	O1—C9—H9B	109.5
C3—O1—C9	117.53 (15)	H9A—C9—H9B	109.5
C2—C1—C6	117.71 (18)	O1—C9—H9C	109.5
C2—C1—C7	122.00 (16)	H9A—C9—H9C	109.5
C6—C1—C7	120.29 (16)	H9B—C9—H9C	109.5
C1—C2—C3	121.26 (17)	N2—C10—C15	118.92 (16)
C1—C2—H2A	119.4	N2—C10—C11	121.93 (16)
C3—C2—H2A	119.4	C15—C10—C11	119.14 (16)
C4—C3—O1	124.23 (18)	C12—C11—C10	119.85 (17)
C4—C3—C2	120.60 (17)	C12—C11—H11	120.1
O1—C3—C2	115.17 (16)	C10—C11—H11	120.1
C3—C4—C5	118.30 (19)	C13—C12—C11	120.15 (17)
C3—C4—H4	120.8	C13—C12—H12	119.9
C5—C4—H4	120.8	C11—C12—H12	119.9
C6—C5—C4	121.90 (18)	C12—C13—C14	120.89 (16)
C6—C5—H5	119.0	C12—C13—N3	119.38 (17)
C4—C5—H5	119.0	C14—C13—N3	119.73 (18)
C5—C6—C1	120.22 (18)	C15—C14—C13	119.09 (18)
C5—C6—H6	119.9	C15—C14—H14	120.5
C1—C6—H6	119.9	C13—C14—H14	120.5
N1—C7—C1	115.80 (16)	C14—C15—C10	120.86 (17)
N1—C7—C8	123.50 (18)	C14—C15—H15	119.6
C1—C7—C8	120.69 (16)	C10—C15—H15	119.6
C7—C8—H8A	109.5		
			
C7—N1—N2—C10	−179.48 (14)	C6—C1—C7—C8	−166.53 (19)
C6—C1—C2—C3	−1.2 (3)	N1—N2—C10—C15	−177.60 (15)
C7—C1—C2—C3	178.84 (16)	N1—N2—C10—C11	3.4 (2)
C9—O1—C3—C4	−3.0 (3)	N2—C10—C11—C12	−179.86 (15)
C9—O1—C3—C2	176.82 (17)	C15—C10—C11—C12	1.2 (3)
C1—C2—C3—C4	0.9 (3)	C10—C11—C12—C13	−0.5 (3)
C1—C2—C3—O1	−178.98 (16)	C11—C12—C13—C14	−0.7 (3)
O1—C3—C4—C5	179.96 (18)	C11—C12—C13—N3	179.40 (16)
C2—C3—C4—C5	0.1 (3)	O2—N3—C13—C12	0.4 (3)
C3—C4—C5—C6	−0.7 (3)	O3—N3—C13—C12	−179.01 (17)
C4—C5—C6—C1	0.3 (3)	O2—N3—C13—C14	−179.54 (17)
C2—C1—C6—C5	0.7 (3)	O3—N3—C13—C14	1.1 (3)
C7—C1—C6—C5	−179.42 (18)	C12—C13—C14—C15	1.2 (3)
N2—N1—C7—C1	179.54 (13)	N3—C13—C14—C15	−178.92 (16)
N2—N1—C7—C8	−0.8 (3)	C13—C14—C15—C10	−0.5 (3)
C2—C1—C7—N1	−166.90 (16)	N2—C10—C15—C14	−179.69 (15)
C6—C1—C7—N1	13.2 (2)	C11—C10—C15—C14	−0.7 (3)
C2—C1—C7—C8	13.4 (3)		

### Hydrogen-bond geometry (Å, °)

**Table:**

*D*—H···*A*	*D*—H	H···*A*	*D*···*A*	*D*—H···*A*
N2—H2···O3^i^	0.86	2.45	3.279 (2)	161

Symmetry codes: (i) *x*−1, −*y*+3/2, *z*−1/2.
